# Ensemble-Based Approaches Ensure Reliability and Reproducibility

**DOI:** 10.1021/acs.jcim.3c01654

**Published:** 2023-11-15

**Authors:** Shunzhou Wan, Agastya P. Bhati, Alexander D. Wade, Peter V. Coveney

**Affiliations:** † Centre for Computational Science, Department of Chemistry, University College London, London WC1H 0AJ, U. K; ‡ Advanced Research Computing Centre, University College London, London WC1H 0AJ, U.K.; § Institute for Informatics, Faculty of Science, University of Amsterdam, 1098XH Amsterdam, The Netherlands

## Abstract

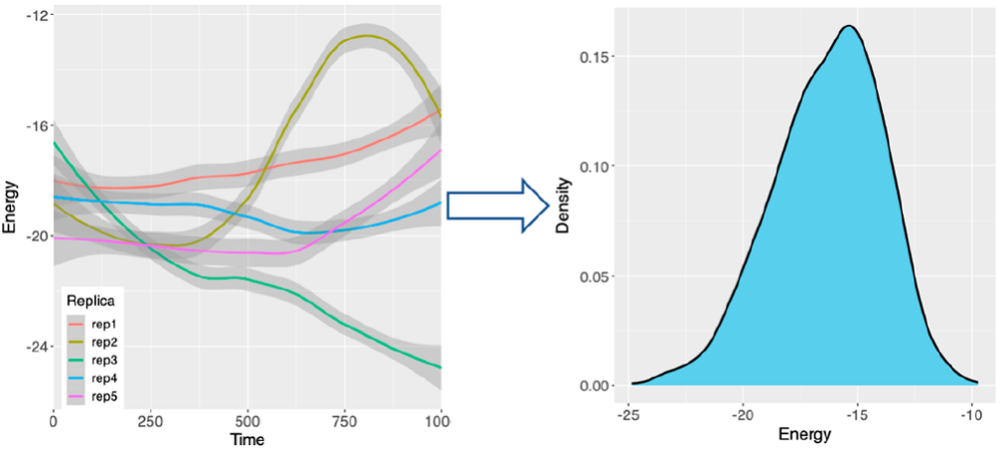

It is increasingly widely recognized that ensemble-based approaches are required to achieve reliability, accuracy, and precision in molecular dynamics calculations. The purpose of the present article is to address a frequently raised question: what is the optimal way to perform ensemble simulation to calculate quantities of interest?

In a recent Editorial on “Guidelines for Reporting Molecular Dynamics Simulations in JCIM Publications”,^1^ the editors put forward recommended guidelines concerning the manner in which classical molecular dynamics (MD) simulations are performed which are important to the scientific community in general and computational chemistry in particular. We have demonstrated^2^ that the MD method exhibits an intrinsically chaotic nature and hence is prone to produce unreliable or unreproducible results. We are therefore obliged to use a *probabilistic* representation for all quantities of interest (QoIs) computed using the method. One point in the JCIM editors’ checklist is “Replica simulations and convergence”, a concept we have been advocating for several years. JCIM now requires that studies reporting on MD simulations should include “at least three replica copies”. Indeed, the common practice in many experimental procedures, and to some extent now at last catching on in molecular simulation, is to perform “three repeats” so that one can estimate the first and second moments of the underlying statistical probability distribution, namely the mean and variance respectively of a QoI. This requirement turns on the assumption that distributions are normal, but while the first two moments completely characterize a normal distribution, more moments are required to characterize a non-normal distribution. We explain below why three measurements are not acceptable in general and recommend against using them as a standard.

Studies have reported non-Gaussian behavior for different QoIs in various applications.^3−8^ In the context of MD simulations, we have reported on numerous occasions the observation of non-Gaussian distributions in binding free energies calculated from both equilibrium^9−14^ and nonequilibrium^15^ approaches. The observation of non-Gaussian distributions from simulations led to the investigation of exceptionally extensive experimental data the results of which we published recently in JCIM.^14^ The distributions of experimental binding free energies exhibit non-normal properties as well for the compounds reported.^14^

A question frequently raised is what is the optimal way to perform MD-based calculation of one or more QoIs? To illustrate the general situation, we select binding free energy as the QoI to answer the above question. It must be pointed out that our findings are in no way exclusively applicable to this case. In materials science, for example, we have demonstrated their applicability just as convincingly as in biomolecular simulations.^11^ We investigate the distributions of calculated binding free energies and test different ensemble simulation protocols while holding the computational resources constant. Suppose we have 60 ns of simulation time available for one compound. What is the most appropriate way to divide these 60 ns to get the most reliable binding free energy estimations? Is it 1 × 60 ns, 6 × 10 ns, 12 × 5 ns, 20 × 3 ns, or 60 × 1 ns runs?

## Non-Gaussian Distributions

**Figure 1:**
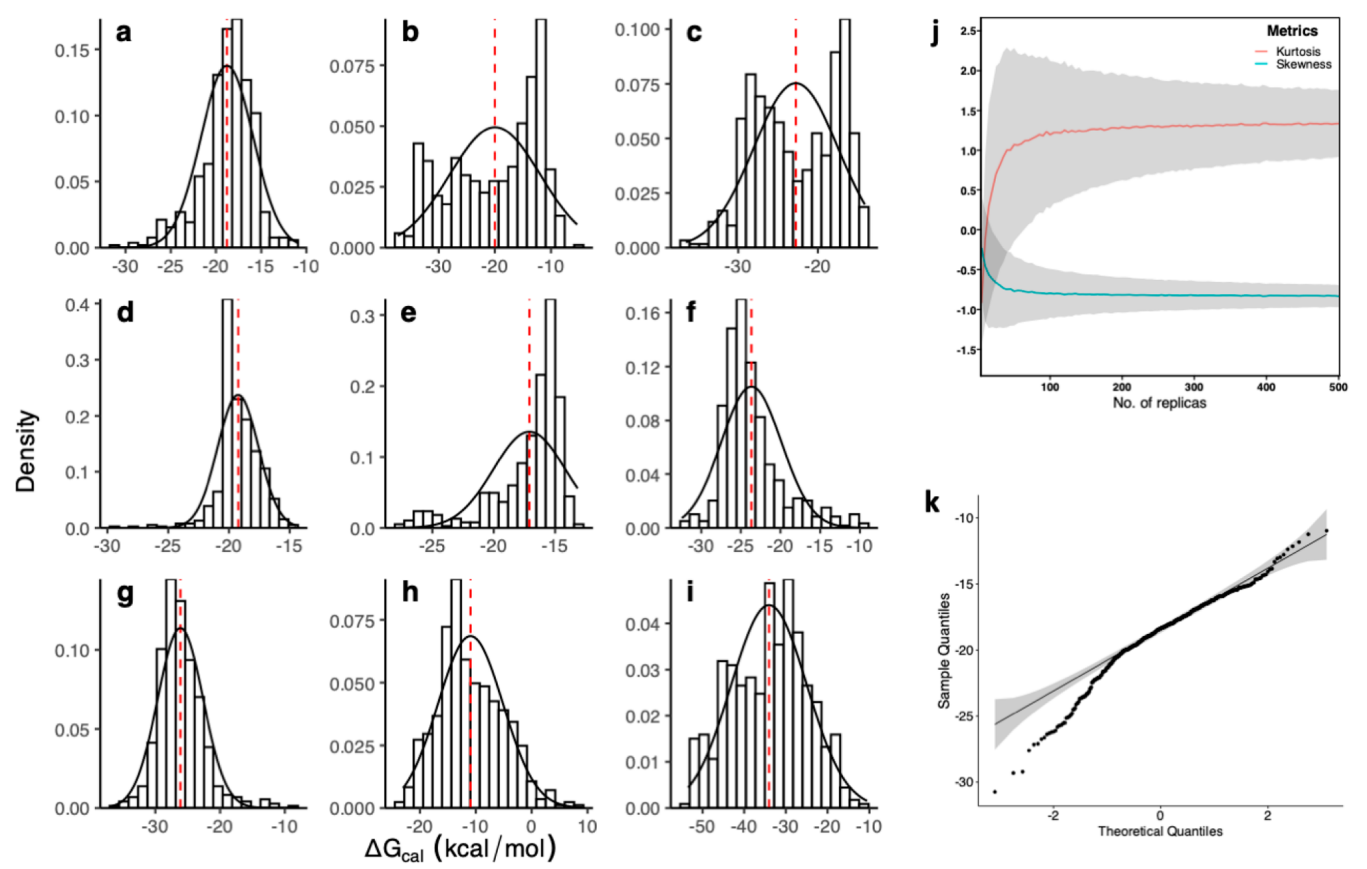
Non-Gaussian characteristics of predicted binding free energies. The distributions of binding free energies (Δ*G*) are obtained from 500-replica ensembles for nine ligand-protein complexes (a-i). The best-fit Gaussian distributions are shown by black solid lines, while the red dashed lines indicate average values. The convergence of the skewness and excess kurtosis (j), with means (solid lines) and standard errors of the mean (shaded region), is shown for one of the ligand-protein complexes investigated (a). The quantile-quantile (Q-Q) plot (k) shows that the quantiles (dots) substantially deviate from an ideal Q-Q plot from a normal distribution (line with shadow showing 95% confidence interval).

In a typical binding free energy study using ESMACS (enhanced sampling of molecular dynamics with approximation of continuum solvent) protocol,^16,17^ we found that the free energy distributions reject the null hypothesis of a normal distribution for >20% of the 400 ligand-protein complexes studied.^11^ The conclusion, however, is not definitive for some molecular systems even from 25-replica ensembles.^18^ To provide conclusive proof of the nature of the distributions, we selected nine complexes from the data set, labeled as “a” to “i” in Figure 1 and Table 1, and increased the number of replicas to 500.

**Table 1:** Skewness and Excess Kurtosis of the Calculated Binding Free Energy Distributions and the Confidence (*p*-Value) That the Null Hypothesis Is False from Shapiro-Wilk and D’Agostino/Pearson Normality Tests^a^

Complex	Skewness	Kurtosis	*p*-value (Shapiro-Wilk)	*p*-value (Pearson)
a	–0.84 [−1.11, −0.57]	1.36 [0.50, 2.14]	5.58 × 10^–11^	1.60 × 10^–14^
b	–0.43 [−0.57, −0.29]	–1.18 [−1.39, −1.03]	3.46 × 10^–16^	5.59 × 10^–64^
c	–0.15 [−0.30, 0.00]	–1.18 [−1.36, −1.03]	6.03 × 10^–13^	1.17 × 10^–60^
d	–0.87 [−1.72, −0.24]	4.84 [2.28, 8.47]	2.96 × 10^–15^	8.90 × 10^–25^
e	–1.73 [−1.93, −1.50]	2.57 [1.39, 3.51]	3.16 × 10^–24^	1.90 × 10^–36^
f	1.27 [1.03, 1.50]	2.03 [1.15, 2.78]	8.48 × 10^–18^	2.05 × 10^–25^
g	1.07 [0.68, 1.53]	3.08 [1.70, 4.55]	2.51 × 10^–13^	2.07 × 10^–24^
h	0.44 [0.26, 0.63]	–0.14 [−0.59, 0.28]	6.65 × 10^–6^	4.21 × 10^–4^
i	–0.10 [−0.24, 0.03]	–0.69 [−0.89, −0.52]	9.62 × 10^–5^	5.25 × 10^–6^

a Errors of the skewness and kurtosis are given in brackets, calculated at the 95% confidence interval using bootstrapping.

The distributions of the predicted absolute binding free energies (ABFE) are summarized graphically in Figure 1. The probability plots manifest the following: 1) differences between means and modes, 2) skewness, 3) kurtosis, 4) long and fat tail(s), and 5) the presence of multimodal distributions. The convergence of skewness and excess kurtosis with the number of replicas is also definitive, showing the two quantities unambiguously deviating from 0 in ensemble simulations with a sufficiently large number of replicas (Figure 1j). The skewness and excess kurtosis are definitively nonzero from 500-replica simulations for most of the systems studied here (Table 1). The Shapiro-Wilk and D’Agostino/Pearson normality tests unequivocally reject the normal null hypothesis for all 9 systems with very high confidence. These statistics require a very large ensemble size to provide a cast-iron answer. The need for such large quantities of data was pointed out by Succi and Coveney.^19^

## Optimal Ensemble Size

Our standard ESMACS protocol employs an ensemble of 25 replicas, with each replica undergoing a 4-ns production run.^16,17^ Our extensive studies over several years demonstrate good convergence and reproducibility from these protocols.^11,16^ When computational resources are limited, as they often are, one may be obliged to “cut corners” on these rigorous protocols.^20^ One would like to know whether to reduce the ensemble size, the temporal duration of the simulation, or a combination of both. Here, we revisit one of our recent simulation studies,^21^ by selecting a subset of ensembles and/or a reduced duration of production runs. The “12×5 ns” protocol, for example, resamples 12 randomly selected replicas and uses only the first 5 ns trajectories to calculate the binding free energies. Many studies have shown that single simulations are not reproducible,^2,6,9,22,23^ while a 1-ns production run is usually too short to produce converged results. We therefore exclude the 1 × 60 ns and 60 × 1 ns options.

**Figure 2:**
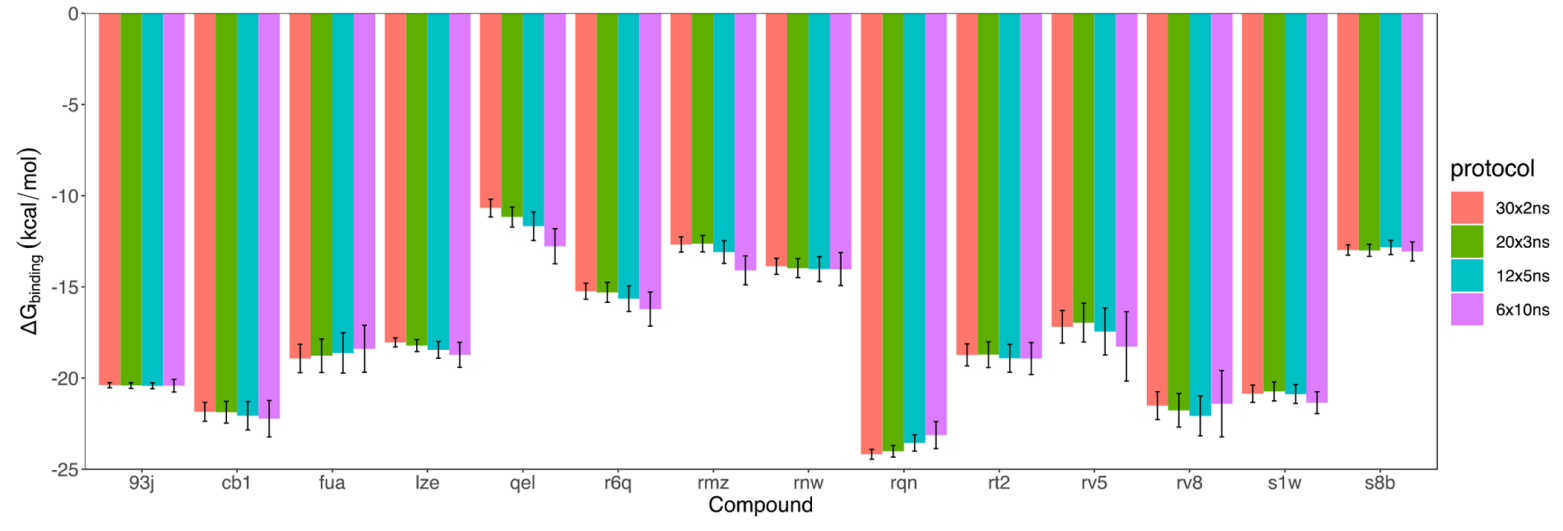
Binding free energies calculated from different protocols. Bootstrapped errors, given to 67% confidence, are provided for the predicted energies.

Figure 2 illustrates our findings. Several observations may be drawn: 1) the differences between calculated binding free energies from different protocols are not statistically significant for most of the molecular systems investigated; 2) the uncertainties increase when the number of replicas is reduced; 3) the free energies typically exhibit a monotonic increase or decrease when the simulation duration is increased. It is evident that no significant differences are observed for the proposed simulation durations (2, 3, 5, or 10 ns). As large ensemble size and short simulations enjoy the benefit from small error bars and short wall-clock run times, we recommend 30 × 2 ns and 20 × 3 ns protocols in order to maximize sampling for a fixed amount of computational time–captured in the phrase “run more simulations for less time”.

It should be noted that whether “for less time” works will depend both on the QoI one is assessing and the conformational space that needs to be sampled. Sufficient sampling of the relevant conformations is important when the properties are determined by multiple minima corresponding to distinct conformations. To capture these in this manner, one would need to start from ensembles which have replicas not only differing in terms of their initial velocities but also corresponding to different initial spatial structures which represent states near these conformations,^11,24^ a recommendation in line with the Editorial guidelines.^1^ Longer ensemble simulations are needed to capture the temporal and spatial characteristics of molecular systems, such as the process of ligand binding.^25^

To investigate the distributions of relative binding free energies (RBFEs) from alchemical methods, we select a subset of a data set from our original TIES (thermodynamic integration with enhanced sampling) study.^26^ We have extended TIES simulations with ensembles of up to 958 replicas;^18^ the results demonstrate that there is a small but significant non-Gaussian behavior in the distribution for one of the five systems. The negative kurtosis for the system, with a 95% confidence interval, can be observed only for ensembles of around 400 replicas. Based on the small absolute value of this kurtosis, −0.29 [−0.47, −0.08], and the lack of a non-Gaussian signal in other observed distributions, we conclude that the non-Gaussian nature may be less common in RBFEs as compared to ABFEs. One significant distinction between RBFE and ABFE calculations lies in the cancellation of numerous large and fluctuating energy contributions within RBFE. Furthermore, RBFE methods rely on shared common atoms between compound pairs, compelling the compounds to adopt the same binding pose simultaneously. Consequently, multiple modes are rarely present in the RBFE distributions. While we recommend an ensemble of 5 or more replicas in general for TIES simulations,^13,26^ one may begin with 3 replicas if cutting corners is required^20^ and then add more replicas for cases where error bars are greater than a chosen threshold.

## Concluding Remarks

Ensemble simulations are necessary to ensure reliability, accuracy, and reproducibility, enabling us to connect ergodic theory and uncertainty quantification. To provide certification for a verification, validation, and uncertainty quantification (VVUQ) standard practice and to make it simpler to quantify uncertainties, a number of toolkits have been developed. One of these is the open-source EasyVVUQ application,^27^ contained within the VECMA^28^ and SEAVEA^29^ toolkits. When there are limitations on computational resources available, we recommend performing a minimum of 10 replicas for ESMACS-style and 3 replicas for TIES-style protocols. We recommend setting a desired level of precision in terms of a predefined threshold for error bars on predictions (say 0.5 kcal/mol). Initially, all calculations can be performed using the minimal number of replicas suggested here to reduce computational costs. Thereafter, further replicas may be included for those systems that do not satisfy the chosen precision threshold criterion. Following such a stepwise procedure allows one to reduce computational costs without compromising substantially the accuracy and precision of results.

## Data and Software Availability

All input structures and AMBER-format topology files along with the predicted Δ*G* values for the 9 compounds binding to the key proteins of SARS-CoV-2 from 500-replica ESMACS simulations are available at 10.23728/B2SHARE.CDD9F8363F364B5682987CD02520B7E3. The data set for the investigation of optimal ensemble sizes was taken from a previous study, which can be found at 10.23728/b2share.1c42a67a73e9424b8192ba65c81077e1.
